# Anatase TiO_2_ adsorption on 3-aminopropyltrimethoxysilane-modified Al or glass surfaces

**DOI:** 10.1016/j.heliyon.2019.e01734

**Published:** 2019-05-18

**Authors:** Masayoshi Kaneko

**Affiliations:** Enzan Senior High School, 440-1, Enzanmikkaitiba, Koshu, Yamanashi, Japan

**Keywords:** Materials science

## Abstract

Herein, anatase titanium dioxide (TiO_2_) nanoparticles were prepared by boiling anatase TiO_2_ in water without using hydrothermal synthesis. This changed the particle diameter because boiling caused particle collision *via* convection. Substrates were then prepared by assembling anatase nanoparticles on 3-aminopropyltrimethoxysilane (APTMS)-functionalized Al surfaces. The effect of pH on the preparation of anatase nanoparticles was investigated, with results indicating that pH 3 is optimal for anatase adsorption on Al surfaces. The anatase TiO_2_ suspension was thus adjusted to pH 3 using dilute HCl solution, and the Al surface selectively adsorbed anatase nanoparticles. This method enabled the adsorption of anatase TiO_2_ nanoparticles at room temperature, without using a heat source. In addition to Al substrates, this method was also found to be applicable to glass surfaces.

## 1 Introduction

Titanium dioxide (TiO_2_) has a wide range of applications. Nanoscale TiO_2_ is a particularly unique substance because of its physicochemical properties, which have been extensively studied [1, 2] Applications of nanoscale TiO_2_ include photocatalysts, self-sustained fuel cells, biocides, solar cells, oxygen reduction catalysts, and optoelectronic devices. TiO_2_ typically exists in the form of anatase, rutile, or brookite. In photocatalytic studies, anatase was found to be more active than crystalline rutile TiO_2_ [1, 2, 3, 4, 5, 6, 7, 8, 9]. In general, anatase TiO_2_ nanoparticles are formed via hydrothermal synthesis. Coating techniques include the sol–gel method and chemical vapor deposition (CVD) [10, 11]. These methods require high temperatures, and hydrothermal synthesis also requires high pressures. Furthermore, a heat source such as an electric furnace is required to generate the high temperatures required to form coatings of anatase TiO_2_ nanoparticles. Contrastingly, metastable anatase and brookite phases irreversibly transform into the equilibrium rutile phase when heated to temperatures above 600°C–800 °C (1,112°F–1,472°F).

This study aims to develop a method for synthesizing TiO_2_ nanoparticles and coatings on Al substrates and glass surfaces without using high temperatures or pressures. 3-Aminopropyltrimethoxysilane (APTMS) can be easily adsorbed onto the surfaces of glass and Al [12, 13]. This study examines the coating method for Al surfaces according to my previous study [12], which reported that boehmite can be functionalized by APTMS. In this study, anatase TiO_2_ was adsorbed onto Al plates treated with APTMS to form a self-assembled monolayer (SAM) at room temperature without using a heat source, such as an electric furnace [12, 14, 15]. Anatase TiO_2_ nanoparticles were prepared by boiling anatase TiO_2_ with water at 100 °C under atmospheric pressure without using hydrothermal synthesis because boiling causes particle collision *via* convection. Particle diameter was reduced by increasing the boiling time, and the preparation of anatase TiO_2_ nanoparticles at low temperatures could effectively avoid some negative effects associated with high temperatures. The method described herein does not require high temperatures and can also be used to adsorb anatase TiO_2_ onto APTMS-functionalized glass surfaces. For example, adsorb anatase TiO_2_ nanoparticles can be adsorbed onto glass windows in self-cleaning processes.

## 2 Experimental

### 2.1 Preparation of anatase TiO_2_ substrates

Substrates were prepared by assembling anatase TiO_2_ nanoparticles on APTMS-functionalized Al plates [12]. To form the nanoparticles, an aqueous anatase TiO_2_ (Titanium (IV) oxide anatase purchased from Kanto Kagaku, Japan) suspension (500 mL, 0.02%) was boiled under vigorous stirring for approximately 20 min and adjusted to pH 3 using dilute HCl solution. The electrostatic potential on a surface, termed its zeta potential, is an important factor in adsorption. Anatase TiO_2_ stably disperses in a strongly acidic medium at pH 2 or less. At low pH values, the nanoparticles remain in a state of dispersion and hydrogen ions are adsorbed on the OH groups, resulting in a positive surface charge, which allows the nanoparticles to be selectively adsorbed at the APTMS sites on the surface. However, the Al surface dissolves at pH 2, so the pH was adjusted pH 3. The Al plate was cleaned with deionized water and heated by boiling in deionized water for approximately 15 min, before being immersed in a 2% (v/v) aqueous APTMS solution for 25 h at room temperature for functionalization. As the APTMS solution is alkaline (pH 11) and dissolves the Al surface, it was first neutralized *via* a silanization treatment using dilute HCl solution. The substrates were then rinsed with ultrapure water and annealed at 110 °C; subsequently, the substrates were immersed in the anatase TiO_2_ suspension for 50 h to allow the assembly of an anatase TiO_2_ nanoparticle film on the silanized Al plate. Furthermore, an aqueous anatase TiO_2_ suspension (500 mL, 0.02%) adsorbed onto the Al plate was used to observe the influence of pH on particle adsorption by adjusting an anatase TiO_2_ suspension to various pH values using dilute HCl solution.

The same procedure was used to adsorb anatase nanoparticles onto the glass surface. To form the nanoparticles, an aqueous anatase TiO_2_ suspension (500 mL, 0.02%) was boiled under vigorous stirring for approximately 20 min and adjusted to pH 2 using a dilute HCl solution. Because anatase TiO_2_ stably disperses in a strongly acidic medium at pH 2 or less. The glass slide was ultrasonically cleaned in a sequential manner in deionized water, isopropyl alcohol, and acetone for 15 min each. Thereafter, the glass slide was soaked in H_2_O:H_2_O_2_(30%): NH_4_OH (5:1:1) for 6 h, and then immersed in a 2% (v/v) aqueous APTMS solution for 25 h at room temperature for functionalization. The slides were further cleaned via sonication in ultrapure water for 20 min and immersed in a 2% (v/v) aqueous APTMS solution for 20 h at room temperature to allow further functionalization [12, 15]. The substrates were then rinsed with ultrapure water, annealed at 110 °C, and subsequently immersed in the anatase TiO_2_ suspension for 50 h to enable the assembly of an anatase TiO_2_ nanoparticle film on the silanized glass slide. APTMS and all other reagents were of analytical reagent grade and used as received from the supplier.

### 2.2 Characterization

Raman spectra were obtained using a Raman spectrophotometer (JASCO NRS-2100), a triple-spectrometer instrument equipped with a holographic notch filter, and a charge-coupled device detector. A solid-state laser (λ = 532 nm) was used for Raman measurements at a power of approximately 19 mW with a spot size of approximately 4 μm in diameter. Scanning electron microscopy (SEM) images were obtained using a field emission microscope (JSM6500F) operated at an accelerating voltage of 15 kV.

## 3 Results and discussion

**Fig. 1:**
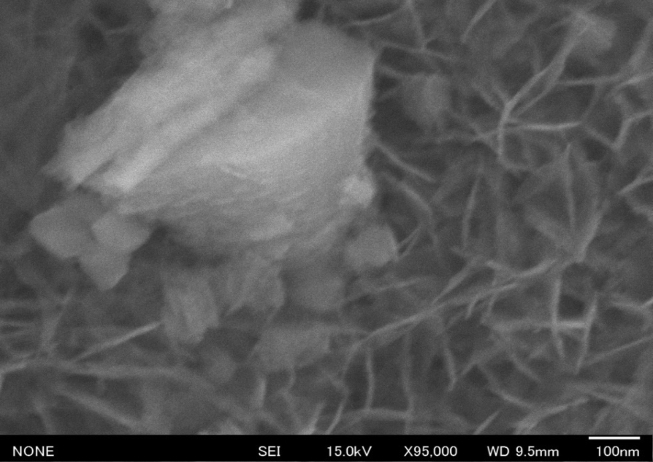
Scanning electron microscopy (SEM) micrographs of the Al surface with adsorbed TiO_2_, coated in aqueous anatase (TiO_2_) solution at a pH 6 (×95000).

**Fig. 2:**
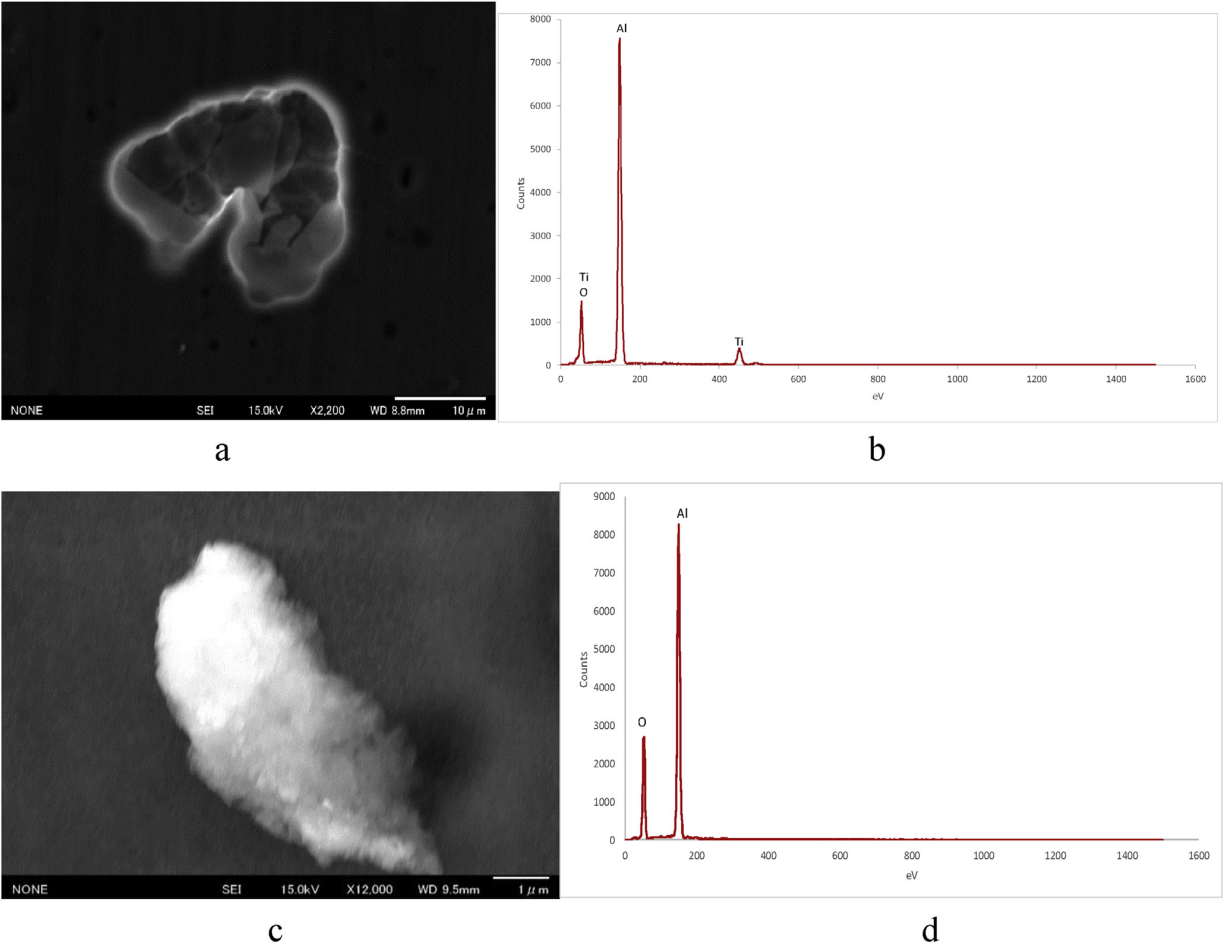
SEM images and point energy dispersive X-ray spectrometry (EDS) analyses of the Al surface coated in aqueous anatase (TiO_2_) solution at a pH 2 (a) (b) and pH 9 (c) (d).

**Fig. 3:**
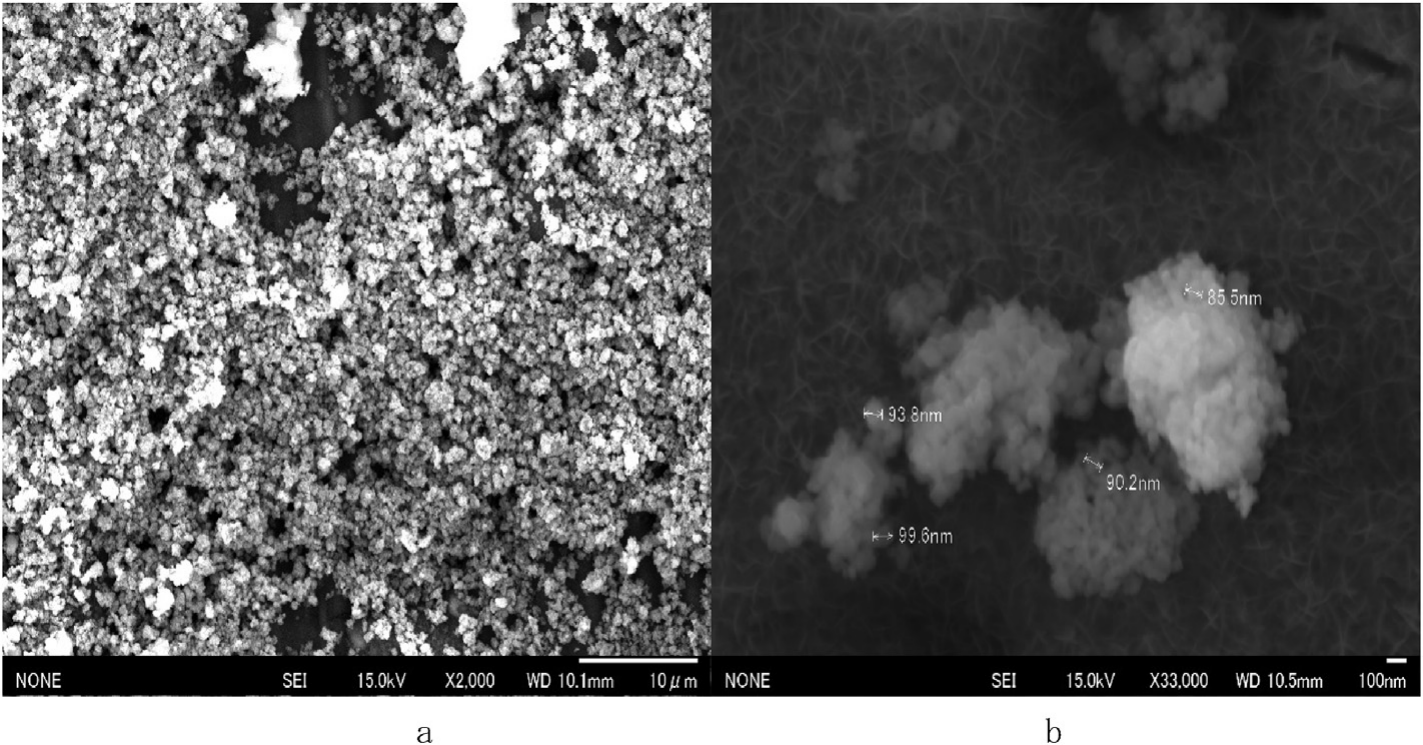
SEM image of the Al surface coated by aqueous anatase (TiO_2_) solution at pH 3. (a) (×2000). (b) (×33000).

Anatase TiO_2_ nanoparticles, with a particle size ranging from 100 to 300 nm, were prepared from anatase TiO_2_ (Kanto Kagaku, Japan). As each particle collided during boiling, the particle size appeared to decrease. The surface of the substrate with anatase was characterized through SEM (Figs. 1, 2, and 3). The SEM observations indicate that an aqueous anatase TiO_2_ solution (500 mL, 0.02%) adsorbed the particles as a function of pH; hence, the solution was adjusted to various pH values using a dilute HCl solution. Note the differences in anatase adsorption between the substrates as pH values of 2, 3, 6, and 9 were used, as shown in Figs. 1, 2, and 3. The observations at pH 2 indicate that TiO_2_ could stably disperse, but the Al surface was slightly dissolved. The anatase TiO2 nanoparticles were not adsorbed on the Al surface at pH 9 owing to the repulsion by the negative static charge.

As reported by Kamiya et al. [16], the electrostatic potential on the anatase TiO_2_ surface, referred to as the zeta potential, is generally positive at low pH values and is either lower or negative at higher pH values. For anatase TiO_2_, the surface isoelectric point was observed at pH 6 [17]. Thus, if the pH is adjusted to be more acidic, hydrogen ions are adsorbed on the OH groups, resulting in a buildup of positive charge. Conversely, under alkaline conditions, OH groups dissociate from the surface, resulting in a buildup of negative charge. Anatase TiO_2_ will be dispersed in a stable manner in strong acidic solutions with pH levels below 2 [15] if these solutions are far from the isoelectric point. Negative charge can be built up at the surface of the APTMS SAM [18].

Fig. 1 shows SEM micrographs of the substrate adsorbed in the aqueous anatase TiO_2_ solution at pH 6. At pH 6, the particles were observed to be coherent.

Fig. 2 shows SEM micrograph and point energy dispersive X-ray spectrometry (EDS) analyses for the substrate at pH 2 and pH 9. The observations at pH 2 indicate that TiO_2_ could stably disperse. Fig. 2(a) shows the Al surface at pH 2. As shown in Fig. 2(b), Ti is adsorbed, but the Al surface is slightly dissolved. Fig. 2(b) shows the EDS analysis, wherein a Ti peak can be observed. As shown Fig. 2(d), the anatase TiO_2_ nanoparticles were not adsorbed on the Al surface at pH 9 owing to repulsion by the negative static charge induced by OH dissociation and because there was a buildup of negative charge on the surface of the APTMS SAM.

The results show that the particles are kept in a state of dispersion at a pH value of 2 or lower as hydrogen ions are adsorbed on the OH groups, creating a positive charge on the surface. Consequently, the anatase TiO_2_ particles are adsorbed at the APTMS sites because they do not cohere. pH 3 was found to be optimal to adsorb anatase TiO_2_ onto Al surfaces. Fig. 3 shows an SEM micrograph of the substrate at pH 3, indicating that the nanoparticles were selectively adsorbed. Particles of nearly equal sizes formed dispersed deposits on the Al surface. The APTMS SAM formed on boehmite indicates that APTMS SAM can attract positively charged TiO_2_ nanoparticles. The Al surface was dissolved at pH 2, i.e., it cannot maintain a normal surface state, however, this is not the case at pH 3. Therefore, the solution was adjusted by limiting it to pH 3. Additionally, TiO_2_ builds up positive charge at a pH value of 5 or lower; thus, pH 3 is the optimal pH for maintaining a strong positive charge of TiO_2_ and a stable Al surface state. At low pH values, nanoparticles remain in a state of dispersion and hydrogen ions are adsorbed onto OH groups, resulting in a positive surface charge allowing the nanoparticles to be selectively adsorbed at APTMS sites on the surface. This is confirmed via SEM imaging.

**Fig. 4:**
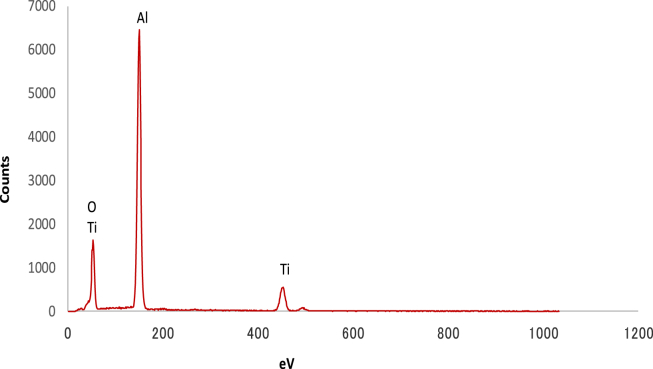
Point EDS analysis of the Al surface coated by aqueous anatase (TiO_2_) solution at pH 3.

**Fig. 5:**
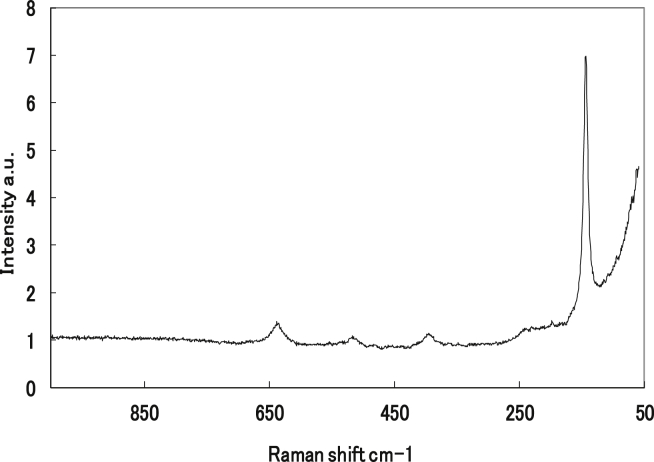
TiO_2_ on Al surface (pH 3). Raman spectrum obtained from the TiO_2_/Al surface. The collection time was 120 s.

The EDS results shown in Fig. 4 reveal that the layer in contact with the Al substrate is TiO_2_. Fig. 5 shows the Raman spectra of the substrates. Raman shifts corresponding to anatase TiO_2_ were observed at 144, 394, 514, and 635 cm^−1^. In contrast, Raman shifts corresponding to rutile (239, 445, and 608 cm^−1^) were not detected [19, 20, 21, 22].

These findings indicate that the anatase phase did not transform to rutile.

**Fig. 6:**
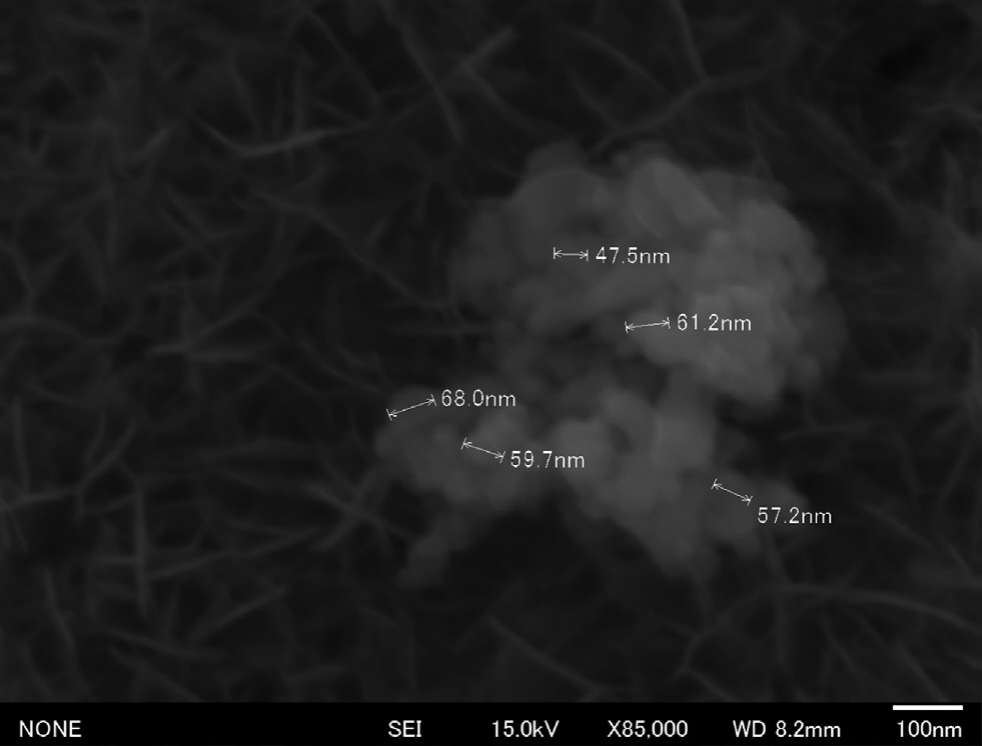
SEM image of the Al surface after boiling in aqueous anatase (TiO_2_) solution under vigorous stirring for 80 min (×85000).

In the experiments conducted herein, the particle size decreased to nanometer scale during boiling under atmospheric pressure. The particle diameter can be changed by increasing the boiling time. Fig. 6 shows an SEM image of the substrate after boiling in an aqueous anatase TiO_2_ solution (500 mL, 0.02%) under vigorous stirring for 80 min. The data indicated that the particles were smaller when boiled for 80 min than when boiled for 20 min. The particle size after boiling for 20 min was approximately 90 nm whereas that after boiling for 80 min was approximately 60 nm. This could be attributed to the possibility of particle collision due to convection, thereby mechanically reducing particle size. Particle diameter could be controlled by varying the boiling time at 100 °C, and smaller particles could be obtained by increasing the boiling duration. These results also showed that this method, involving the preparation of anatase TiO_2_ nanoparticles at relatively low temperatures and coating anatase TiO_2_ nanoparticles on an Al surface at room temperature, did not generate the issues associated with high temperature processes. This method enables the production of a coating of anatase TiO_2_ on an ATMPS-functionalized glass surface. The same effect was noted using a glass substrate at pH 2; however, anatase TiO_2_ NPs were deposited to a lesser extent on glass surfaces at pH 2. The primary amine is ANH_2_. The result regarding boehmite indicates that the protons in the surface AlOAOH groups were detached, resulting in a negatively charged surface capable of attracting positively charged TiO_2_ NPs. Therefore, the anatase TiO_2_ NPs are deposited on the surfaces from pH 2 to pH 3.

**Fig. 7:**
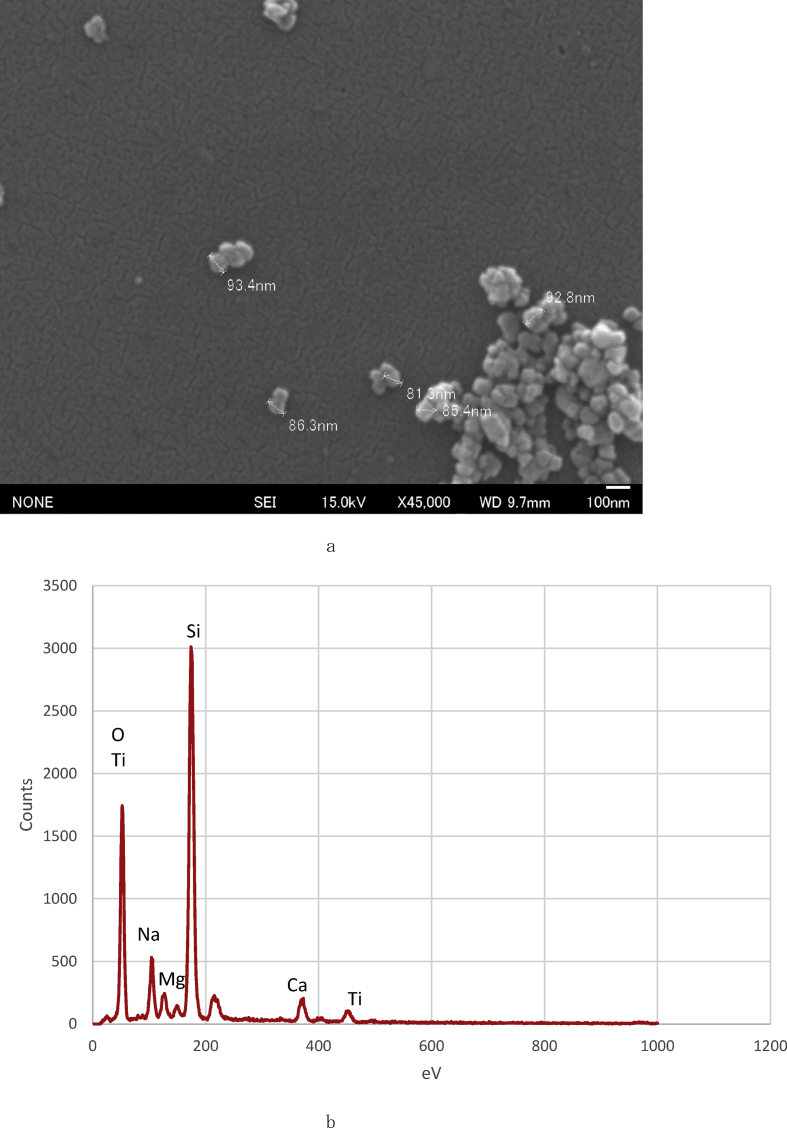
SEM images of the glass surface coated in aqueous anatase (TiO_2_) solution at pH 2 (×45000). (a). Point EDS analysis of the glass surface. (b).

**Fig. 8:**
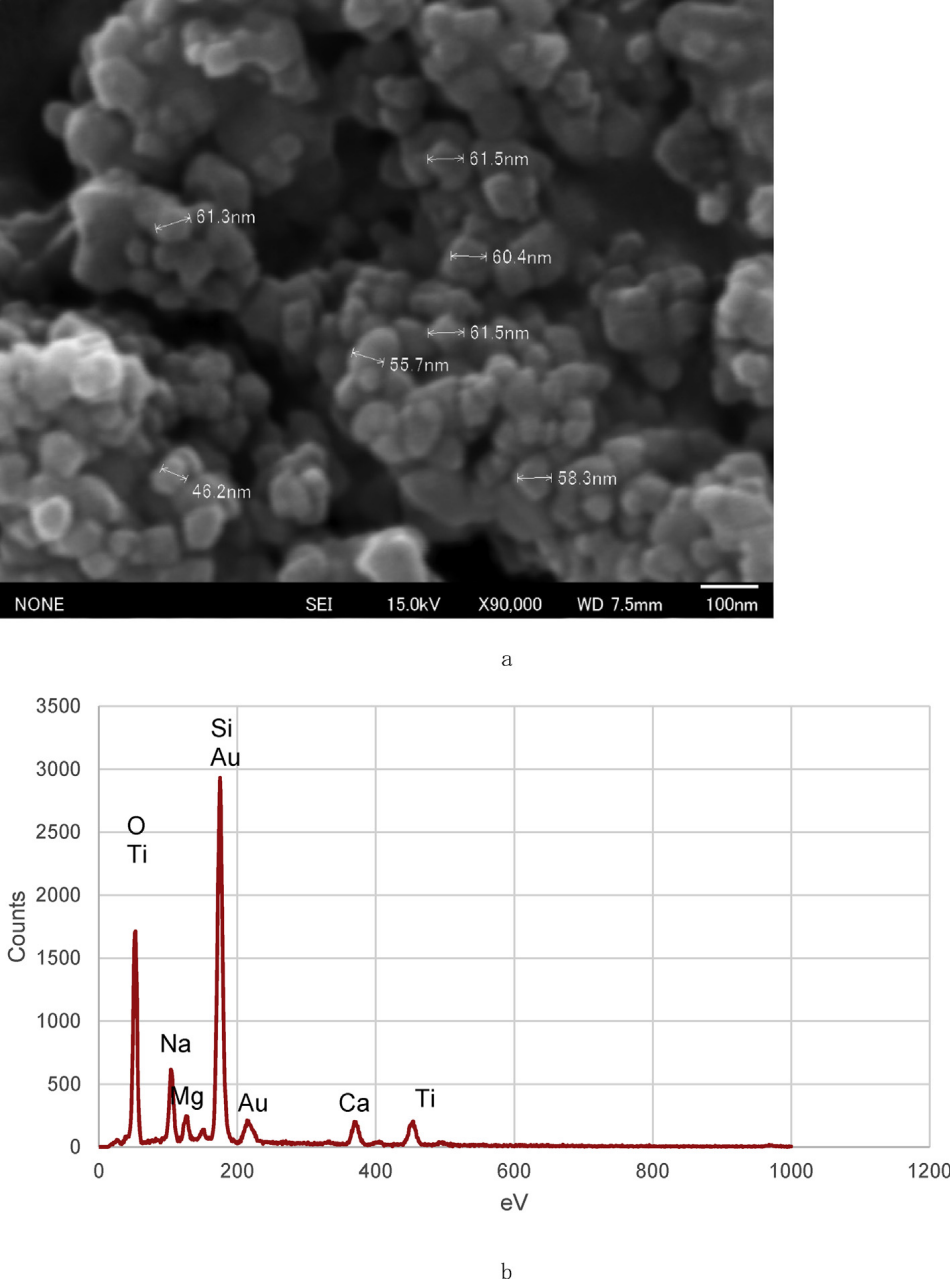
SEM images of the glass surface coated in aqueous anatase (TiO_2_) solution at pH 2 under vigorous stirring for 80 min (×90000). (a) Point EDS analysis of the glass surface. (b).

When boehmite was not used, the protons in the surface SiAOH groups were not detached completely, and thus could not create a partially negatively charged surface. Consequently, this surface is not capable of attracting positively charged TiO_2_ NPs. According to the method proposed by Sung-Te Chen et al [18], if the functionalization of APTMS SAMs is performed in a basic solution for more than 30 s, the surface may deposit more TiO_2_ NPs; however, this phenomenon was not explored in the experiment reported herein. Fig. 7(a) shows the SEM image of anatase TiO_2_ nanoparticles on a glass surface, and Fig. 7(b) shows the corresponding point EDS analysis. Additionally, Fig. 8(a) shows an SEM image of the substrate after boiling in an aqueous anatase TiO_2_ solution (500 mL, 0.02%) under vigorous stirring for 80 min, and Fig. 8(b) shows the corresponding point EDS analysis. The particle size after boiling for 20 min was approximately 90 nm and that after boiling for 80 min was approximately 60 nm. This result suggests that particles of submicron size reduced nanoparticle size by boiling at atmospheric pressure.

## 4 Conclusions

The method outlined herein aimed to change particle size to prepare anatase TiO_2_ nanoparticles from anatase TiO_2_ particles without using hydrothermal synthesis. Nanoparticles were formed at 100 °C while controlling the solvent's pH. They were prepared by boiling an aqueous anatase TiO_2_ suspension (500 mL, 0.02%) under vigorous stirring for 20 min. Following this, HCl was added to induce a stable nanoparticle dispersion. The electrostatic potential on a surface, termed its zeta potential, is an important factor in adsorption. Anatase TiO_2_ stably disperses in a strongly acidic medium at pH 2 or less, although the Al surface dissolves at this pH. Most particles are expected to cohere at pH 6. As the surface of APTMS SAM builds up negative charge, the nanoparticles were not adsorbed on the Al surface at pH 9 because of repulsion from negative static charges formed as the OH groups dissociated from the surface. The Al surface slightly dissolved at pH 2, but not at pH 3. Anatase TiO_2_ would therefore be stably dispersed in a strong acidic solution, with pH 2 and pH 3 being the nearest pH in the range of pH ≤ 5. pH 3 was determined to be optimal for anatase TiO_2_ adsorption on the Al surface. Regarding the glass surface, pH 2 was considered to be optimal to adsorb anatase TiO_2_, as the particles are kept in a state of dispersion at pH values below 2. At low pH values, the nanoparticles remain in a state of dispersion and hydrogen ions are adsorbed on the OH groups, resulting in a positive surface charge, which allows the nanoparticles to be selectively adsorbed at the APTMS sites on the surface, as confirmed through SEM imaging. Using this method, anatase TiO_2_ nanoparticles could be easily prepared at low temperatures and adsorbed onto Al surfaces at room temperature (an electric furnace was not required). With respect to submicron particles, the particle diameter could be controlled at the low temperature of 100 °C used in this process. Based on these results, the proposed method can be used to avoid the undesirable effects associated with high temperatures.

## Declarations

### Author contribution statement

Masayoshi Kaneko: Conceived and designed the experiments; Performed the experiments; Analyzed and interpreted the data; Contributed reagents, materials, analysis tools or data; Wrote the paper.

### Funding statement

This research did not receive any specific grant from funding agencies in the public, commercial, or not-for-profit sectors.

### Competing interest statement

The authors declare no conflict of interest.

### Additional information

No additional information is available for this paper.
